# Brucella melitensis-Induced Transaminitis

**DOI:** 10.7759/cureus.13656

**Published:** 2021-03-02

**Authors:** Rajesh Essrani, Anastasia Shnitser

**Affiliations:** 1 Internal Medicine, Lehigh Valley Health Network, Allentown, USA; 2 Gastroenterology and Hepatology, Lehigh Valley Health Network, Allentown, USA

**Keywords:** brucella melitensis, transaminitis, travel health

## Abstract

Brucellosis is infrequently reported in the United States and is mostly an occupational hazard among workers engaged in livestock raising and processing. It is a systemic infectious disease and can involve the liver in varying ways, ranging from benign subclinical increases in serum aminotransferase levels to ominous chronic suppurative disease. It is endemic in many countries, primarily those of the Mediterranean region. It is usually treated with antibiotics. We present a case of a 37-year-old female who developed *Brucella melitensis*-induced transaminitis, which improved with proper diagnosis and management.

## Introduction

Brucellosis is a common zoonotic disease distributed around the world, caused by microorganisms of the genus *Brucella*, an intracellular bacterium that widely affects animals and can cause systemic infections in humans with an acute, subacute, or chronic course [1]. The disease is rare in industrialized countries because of the routine screening of domestic livestock and animal vaccination. Brucellosis is infrequently reported in the United States. It results from contact with fluids from infected animals (sheep, cattle, pigs, dogs, rats and goats) or by ingestion of unpasteurized dairy products (soft cheese, ice cream and raw milk) [2]. It mainly presents with insidious onset of fever, night sweats (associated with a strong, peculiar, moldy odor), weight loss, headache, fatigue, abdominal pain, malaise, and can result in variable hepatic manifestations, ranging from a benign subclinical rise in serum aminotransferase levels to ominous chronic suppurative disease [3]. It requires a specific diagnosis based on epidemiological analysis and confirmatory laboratory tests to initiate the appropriate antibiotic treatment.

This article was presented as a poster at the American College of Gastroenterology (ACG) Virtual ePoster Hall in October 2020.

## Case presentation

**Figure 1:**
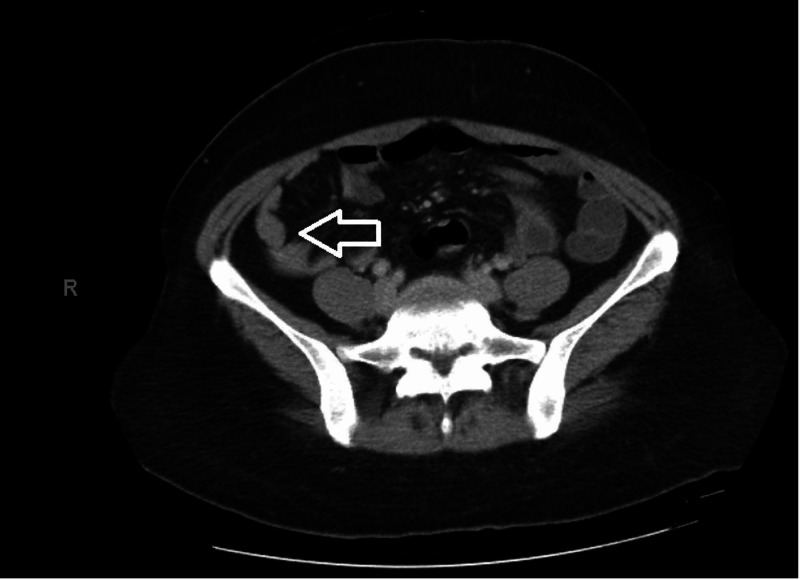
CT abdomen and pelvis showing right colonic wall patchy thickening consistent with colitis

A 37-year-old African American female with no significant past medical history presented to the hospital with fever and severe headaches. Her symptoms initially started with generalized myalgia and joint stiffness; they later on progressed into a fever, night sweats and headaches which led her to come for an evaluation. She immigrated from Somalia 10 years prior and has traveled extensively outside of the United States. She reported no weight loss, sick contact, illicit drug abuse, and personal/family history of tuberculosis. Physical exam on admission was significant for mild right upper quadrant tenderness. Blood work revealed white blood cell count 2.53 K/uL (4.00 - 10.80 K/uL), hemoglobin count 10.1 g/dL (12.0 - 15.3 g/dL), platelet count 131 K/uL (140 - 400 K/uL), creatinine 0.6 mg/dL (0.5 - 1.0 mg/dL), aspartate transaminase 113 U/L (10 - 35 U/L), alanine transaminase 77 U/L (10 - 35 U/L), alkaline phosphatase 134 U/L (0 - 153 U/L), and total bilirubin 1.4 mg/dL (0 - 1.2 mg/dL) (Table 1). Cross-sectional imaging of chest, abdomen and pelvis revealed ground-glass opacities in the left upper lung lobe and right colonic wall patchy thickening consistent with colitis (Figure 1). She had extensive diagnostic workup including bronchoscopy and lumbar puncture, which was initially unremarkable. Blood culture resulted in *Brucella melitensis* after eight days as it is slow growing organism. Additional workup for infectious etiologies such as Cytomegalovirus (CMV), Epstein-Barr virus (EBV), Hepatitis A, B, and C, and tick-borne panel was negative. She was evaluated for drug-induced, autoimmune, and metabolic etiologies of her laboratory abnormalities, which were eventually ruled out. She was subsequently diagnosed with *Brucella melitensis* bacteremia. Initial treatment included doxycycline and gentamycin with symptomatic improvement. She was discharged home on rifampin and doxycycline to complete six weeks of therapy. Additional history revealed that the patient drank camel milk in the United Arab Emirates seven months prior to admission. She followed up as an outpatient with repeat liver enzymes which normalized after completing antibiotic therapy.

**Table 1:** Blood Work

Test	Patient Result	Reference
White Blood Cell count	2.53 K/uL	4.00 - 10.80 K/uL
Hemoglobin Count	10.1 g/dL	12.0 - 15.3 g/dL
Platelet Count	131 K/uL	140 - 400 K/uL
Creatinine	0.6 mg/dL	0.5 - 1.0 mg/dL
Aspartate Transaminase	113 U/L	10 - 35 U/L
Alanine Transaminase	77 U/L	10 - 35 U/L
Alkaline Phosphatase	134 U/L	0 - 153 U/L
Total Bilirubin	1.4 mg/dL	0 - 1.2 mg/dL

## Discussion

Brucellosis is endemic in countries of the Middle East, the Indian subcontinent, Mediterranean basin, Central Asia, sub-Saharan Africa, China, and parts of Mexico and Central and South America [4,5]. The most common way for transmission to humans is the consumption of infected, unpasteurized animal products, contact of mucous membrane or skin with infected animal tissue (such as miscarriage products or placenta) or infected animal fluids (such as urine, milk, or blood), and inhalation of infected aerosolized particles [2,6,7]. Laboratory workers should be informed about the diagnostic possibility of brucellosis in order to implement special culture techniques and appropriate precautions. The incubation period is usually two to four weeks; occasionally, it may be as long as several months. Brucellosis is a systemic disease with a wide clinical spectrum from asymptomatic disease to severe or fatal illness. It mainly presents with insidious onset of fever, night sweats (associated with a strong, peculiar, moldy odor), weight loss, headache, fatigue, abdominal pain, malaise, and arthralgias. Infective endocarditis, although rare, is the most devastating complication from systemic brucellosis and could require surgical intervention. Physical findings are variable and nonspecific; splenomegaly, hepatomegaly, and/or lymphadenopathy may be observed. Brucellosis should be suspected in patients with signs and symptoms of fever, malaise, night sweats, and arthralgia in the setting of epidemiologic exposure (consumption of unpasteurized dairy products, animal exposure in an endemic area, and/or occupational exposure). A definitive diagnosis may be made by culture of the organism (from blood, body fluids, or tissue), or ≥ a four-fold rise in Brucella antibody titer between acute and convalescent-phase serum specimens obtained ≥two weeks apart [8]. In our case, appropriate history, clinical signs/symptoms, diagnostic images, and laboratory findings ruled out other possible causes of acute liver injury, leading to the diagnosis of *Brucella melitensis*-induced transaminitis. The standard treatment for brucellosis includes the use of antibiotics with activity in acidic intracellular environments (such as doxycycline and rifampin), use of combination therapy (given high relapse rates with monotherapy), and prolonged duration of treatment. There are no vaccines for the prevention of brucellosis in humans. Patients should be counseled to wait until the completion of therapy before unprotected sexual contact, and lactating women should be advised to discontinue breastfeeding until completion of treatment [9].

## Conclusions

The clinical presentation of brucellosis can be very imprecise because it can affect any organ system. It should be considered in the differential diagnosis of fever and transaminitis for those who live or have visited endemic areas. We recommend obtaining a careful occupational and travel history of patients who have transaminitis. It is usually diagnosed with blood culture or antibody titers along with labs like complete blood count and liver function tests. The incubation period is usually two to four weeks; occasionally, it may be as long as several months as it is very slow to grow compared to *Staphylococcus aureus* so it is important to keep this in mind.

## References

[1] Franco MP, Mulder M, Gilman RH, Smits HL (2007). Human brucellosis. Lancet Infect Dis.

[2] de Figueiredo P, Ficht TA, Rice-Ficht A, Rossetti CA, Adams LG (2015). Pathogenesis and immunobiology of brucellosis: review of Brucella-host interactions. Am J Pathol.

[3] Akritidis N, Tzivras M, Delladetsima I, Stefanaki S, Moutsopoulos HM, Pappas G (2007). The liver in brucellosis. Clin Gastroenterol Hepatol.

[4] Pappas G, Papadimitriou P, Akritidis N, Christou L, Tsianos EV (2006). The new global map of human brucellosis. Lancet Infect Dis.

[5] Seleem MN, Boyle SM, Sriranganathan N (2010). Brucellosis: a re-emerging zoonosis. Vet Microbiol.

[6] Franco MP, Mulder M, Gilman RH, Smits HL (2007). Human brucellosis. Lancet Infect Dis.

[7] Bosilkovski M, Dimzova M, Grozdanovski K (2009). Natural history of brucellosis in an endemic region in different time periods. Acta Clin Croat.

[8] Andonopoulos AP, Asimakopoulos G, Anastasiou E, Bassaris HP (1986). Brucella arthritis. Scand J Rheumatol.

[9] Tuon FF, Gondolfo RB, Cerchiari N (2017). Human-to-human transmission of Brucella - a systematic review. Trop Med Int Health.

